# Role of glyoxalase 1 in methylglyoxal detoxification–the broad player of psychiatric disorders

**DOI:** 10.1016/j.redox.2021.102222

**Published:** 2021-12-22

**Authors:** Kazuya Toriumi, Mitsuhiro Miyashita, Kazuhiro Suzuki, Koichi Tabata, Yasue Horiuchi, Hiroaki Ishida, Masanari Itokawa, Makoto Arai

**Affiliations:** a Schizophrenia Research Project, Department of Psychiatry and Behavioral Sciences, Tokyo Metropolitan Institute of Medical Science, Tokyo, 156-8506, Japan; b Department of Psychiatry, Tokyo Metropolitan Matsuzawa Hospital, Setagaya-ku, Tokyo, 156-0057, Japan; c Department of Psychiatry, Takatsuki Hospital, Hachioji, Tokyo, 192-0005, Japan; d Department of Psychiatry, Graduate School of Medicine, Shinshu University, Nagano, 390-8621, Japan; e Department of Psychiatry and Behavioral Science, Tokyo Medical and Dental University Graduate School, Tokyo, Japan

**Keywords:** Glyoxalase 1, Methylglyoxal, Anxiety, Depression, Autism, Schizophrenia

## Abstract

Methylglyoxal (MG) is a highly reactive α-ketoaldehyde formed endogenously as a byproduct of the glycolytic pathway. To remove MG, various detoxification systems work together *in vivo*, including the glyoxalase system, which enzymatically degrades MG using glyoxalase 1 (GLO1) and GLO2. Recently, numerous reports have shown that GLO1 expression and MG accumulation in the brain are involved in the pathogenesis of psychiatric disorders, such as anxiety disorder, depression, autism, and schizophrenia. Furthermore, it has been reported that GLO1 inhibitors may be promising drugs for the treatment of psychiatric disorders. In this review, we discuss the recent findings of the effects of altered GLO1 function on mental behavior, especially focusing on results obtained from animal models.

## 1 Introduction

Methylglyoxal (MG) is a highly reactive α-ketoaldehyde formed endogenously as a byproduct of the glycolytic pathway either by the degradation of triphosphates or by nonenzymatic fragmentation of sugar [1]. MG accumulates under conditions of hyperglycemia, impaired glucose metabolism, or oxidative stress. Excess MG formation causes mitochondrial impairment and reactive oxygen species production, which further increases oxidative stress. MG also reacts with proteins, DNA and other biomolecules, leading to the formation of advanced glycation end products (AGEs) [2,3]. These imply that accumulation of MG causes damage to various tissues and organs [4], resulting in aging and diabetic complications, such as neuropathy, retinopathy, and ischemic heart disease. In addition, AGEs, formed by MG, induce aberrant inflammation by binding to receptors for AGEs, which play a role in chronic inflammation and Alzheimer's disease [5,6].

**Fig. 1:**
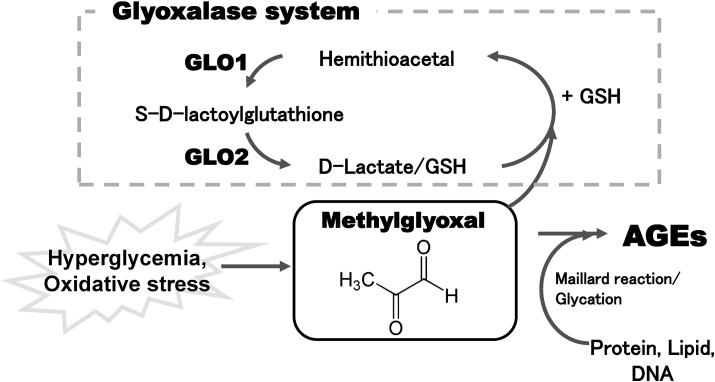
Glyoxalase detoxification system. Accumulation of reactive carbonyl compounds results in the modification of proteins and the eventual formation of advanced glycation end (AGE) products and methylglyoxal-adducts. Glyoxalase proteins are ubiquitously expressed in various tissues, including the brain, and provide an effective defense against the accumulation of reactive dicarbonyl compounds. GLO1, glyoxalase I; GLO2, glyoxalase II; GSH, glutathione.

To remove MG, various detoxification systems work together *in vivo*. The glyoxalase system, comprising two enzymes, glyoxalase (GLO) 1 and GLO2, is an enzymatic pathway that catalyzes the glutathione-dependent detoxification of MG (Fig. 1). GLO1 catalyzes the conversion of the hemithioacetal formed by the non-enzymatic reaction of glutathione with MG to S-d-lactoylglutathione, which is then converted to d-lactate by GLO2 [1,7]. GLO1 and GLO2 are ubiquitously expressed in various tissues, including the brain, and provide an effective defense against MG accumulation. Furthermore, DJ-1, aldo-keto reductase, and aldehyde dehydrogenase are also known as enzymes with MG removal activity, and these enzymes work in concert with GLO1 and GLO2 to protect against MG-induced cytotoxicity [[8], [9], [10], [11], [12]].

To date, studies of the MG detoxification system have mainly been conducted in fields related to physical disorders, such as diabetes and renal dysfunction. However, recent years have shown an increased interest in the role of GLO1 and its clinical applications in mental illness. In the present review, we summarize the latest findings on the effects of altered GLO1 function on mental behavior, focusing on the findings from animal models.

## 2 Clinical studies

A C419A single nucleotide polymorphism (SNP) (reported in dbSNP as *rs2736654* or *rs4746;* NM_006708.3:c.332A>C, p.Glu111Ala) present in *GLO1*, which causes an Ala111Glu substitution in the protein, has been reported to be associated with panic disorder [13] and autism [14,15]. Junaid et al. reported that the GLO1 enzymatic activity in the autopsied brains of patients with autism was 38% lower than that in healthy controls, and an accumulation of AGEs was also found in the brains of autistic patients [14]. Another clinical study also reported that the GLO1 enzymatic activity was decreased in autistic patients with the A419 allele [16]. Interestingly, another study found that the C419A SNP might affect alternative splicing in GLO1, probably leading to a change in GLO1 enzymatic activity [17].

However, contrary to these reports, some clinical studies have demonstrated no association between C419A SNP and autism [[18], [19], [20]]. One clinical study even reported an association between the C419 allele, instead of A419, and autism, and found lower GLO1 enzymatic activity in autistic patients with the C419 allele than in those with A419 [21]. Consistent with these findings, our research showed that GLO1 enzymatic activity was decreased in schizophrenia patients with the C419 allele, compared with those with A419, although there is no significant change in enzymatic activity between GLO1 with A419 and with C419 using recombinant proteins [22]. We have reported that several patients with schizophrenia have a novel heterozygous frameshift P122fs (*rs754100427*; NP_006699.2:p.Pro122fs) and an A419C SNP in *GLO1*, resulting in 40–50% and 15–20% reductions in enzymatic activity, respectively [22]. The frameshift mutation P122fs has also been reported by another group [23]. Moreover, synchrotron radiation nanotomography of the structures of cerebral tissues of the anterior cingulate cortex revealed that a schizophrenia patient with the frameshift P122fs in *GLO1* showed marked differences in the curvature of neurites, compared with healthy controls [24]. Another group recently reported that significant differences in *GLO1* mRNA expression and enzymatic activity were found in the peripheral blood of first-onset antipsychotic-naïve patients with schizophrenia and controls, and that receiver operating characteristic (ROC) curve analysis showed that GLO1 could predict schizophrenia risk (mRNA, P = 4.75 × 10^−6^; enzymatic activity, P = 1.43 × 10^−7^) [25]. A clinical study found MG accumulation in the peripheral blood of a subgroup of patients with schizophrenia [26]. These findings suggest that the decrease in GLO1 activity and subsequent accumulation of MG are involved in the pathogenesis of schizophrenia. Additionally, reduced *Glo1* mRNA expression was also observed in major depressive and bipolar disorder patients in a current depressive state, whereas no correlation was observed in a remissive state [27]. This indicated that *Glo1* expression level was inversely proportional to the severity score on the Hamilton Depression Rating Scale, suggesting that decreased *GLO1* expression might be related to pathophysiology of depression disorder.

However, it is also important to note that recent genome-wide association studies (GWASs) have not shown single nucleotide variants (SNVs) near the *GLO1* gene as being relevant to psychiatric diseases. A genome-wide meta-analysis demonstrated the association between an SNV in the *GLO1* gene and major depression disorder in males, but there was no genome-wide significance [28]. It should be noted that the results of the clinical studies described above are based on a very limited number of samples, and the results of the genetic analysis of *GLO1* may be false positives. In fact, GWASs, case-control studies, and meta-analysis studies have reported no association between SNVs in the *GLO1* gene and psychiatric disorders [18,20,29].

We have yet to obtain consistent results regarding the relationship between SNVs in *GLO1* and psychiatric disorders. However, the finding that GLO1 enzymatic activity is decreased in patients with psychiatric disorders is likely to be consistent. For example, it is necessary to consider not only the SNVs in *GLO1* and its gene expression, but also the effects of environmental factors, including levels of zinc as a cofactor [30] and phosphorylation of T107 in GLO1 [31], and treatment with psychotropic drugs on the enzymatic activity of GLO1. Thus, rather than discussing the SNVs, it is more important to discuss the relationship between reduced GLO1 activity and psychiatric disorders.

## 3 Animal models

### 3.1 Anxiety

A number of studies have reported an association between *Glo1* expression and anxiety-like behavior in rodents. Table 1 summarizes the findings of mouse models in which *Glo1* expression has been experimentally manipulated. First, using a combination of behavioral analysis of six inbred mouse strains and quantitative gene expression, Hovatta et al. demonstrated that there was a statistically significant positive correlation between anxiety-like behavior and *Glo1* expression as well as GLO1 enzymatic activity in the brain [32]. Moreover, *Glo1* overexpression in the cingulate cortex enhanced anxiety-like behavior, whereas *Glo1* knockdown by siRNA reduced anxiety-like behavior. These findings suggested that *Glo1* is a strong candidate for regulating anxiety-like behavior, and a higher expression of GLO1 increased anxiety. The results were replicated in several studies. For example, a whole brain proteomics study showed that the expression of the GLO1 protein is significantly higher in anxious mice [33]. In DBA/2C mice, a highly anxious mouse strain, *Glo1* mRNA showed increased expression in the brain [34]. In addition, a less than 475 kb tandem duplication on chromosome 17, which includes *Glo1* in mice, has been identified, and CD-1 mice carrying the duplication of the *Glo1* region showed higher *Glo1* expression and greater anxiety-like behavior [35]. Overexpression of *Glo1* in mice on a transgenic bacterial artificial chromosome [36] and overexpression of *Glo1* only in neurons displayed enhanced anxiety-like behavior [37]. Consistent with these findings, treatment with a GLO1 inhibitor reduced anxiety-like behavior [36,[38], [39], [40]], as summarized in Table 2. *Glo1* knockout (KO) mice have also been reported to exhibit less anxiety-like behavior [41]. These findings suggest that increased *Glo1* expression in neurons enhances anxiety-like behavior in mice.

**Table 1:** Genetic mouse model for *Glo1* (by Hambsch et al. [87] with some modifications.).

Authors	Animal model		GLO1 expression in brain	GLO1 activity	MG level	AGEs	Behavior	Rescue
Hovatta, et al.[32]	A/J, DBA/2J mouse	High anxiety strain (compared with C57BL/6J, FVB/NJ)	↑ (AMY, CIN, BNS HIP, HYP, PAG)	↑	-	-	Anxiety↑ (OF)	Improved by Glo1 OE in the cingulate cortex
Krömer, et al.[45], Hambsch, et al.[44], Ditzen, et al.[46], Zhang, et al.[47]	CD1 mouse	HAB inbred	↓	-	-	-	Anxiety↑ (EPM, LDB), Locomotor↓, USV↑	-
		LAB inbred	↑	-	↑	MG-dependent protein↑	Anxiety↓ (EPM, LDB), Locomotor↑, USV↓, Depression↓(TS,FS)	-
Williams, et al.[35], Distler, et al.[81]	BXD RI lines	Glo1 gene duplication	↑	-	-	-	Anxiety↑ (OF), High atmospheric pressure-induced seizure↑	-
Szegö, et al.[33]	MG15 inbred mouse	AX inbred	↑	-	-	-	Anxiety↑ (EPM, OF, LDB)	-
Distler, et al.[36, 81], McMurray, et al.[82], Barkley-Levenson, et al.[84]	C57BL/6J, FVB/NJ mouse	Glo1 OE	↑	↑	↓	-	Anxiety↑ (OF), Pilocarpine-induced seizure↓, Alcohol comsumption↑, HIC after alcohol injection↑,	-
McMurray, et al.[37]	C57BL/6J mouse	Glo1 OE in neuron	↑	↑	-	-	Anxiety↑ (OF)	-
Jang, et al.[41]	C57BL/6N mouse	Glo1 KO	↓	↓	-	MG-H1 n.s.	Anxiety↓ (OF, LDB), Depression-like behavior↑ (FS), Locomotor↑(OF)	-
McMurray, et al.[39, 82]	C57BL/6J mouse	Glo1 hemizygous KD	-	↓ [88]	-	-	Depression↓(TS, FS), Alcohol comsumption↓	-
Matsuo, et al.[34]	DBA/2C mouse	High anxiety strain (compared with C57BL/6J)	↑ (AMY, HIP, THA, HYP, STR, CER)	-	-	-	Anxiety↑ (EPM, OF), Locomotor↓	-
Toriumi, et al.[58]	C57BL/6J mouse	Glo1 KO	↓	↓	n.s.	-	Depression↓ (FS)	-
	C57BL/6J mouse	Glo1 KO + VB6 deficiency	-	-	↑ (PFC, HIP, STR), n.s. (NAC, BS, CER)	↑ (PFC, HIP, NAC, STR, BS, CER)[67]	Depression↓ (FS), Locomotor↓, Sociability↓, Long-term memory↓ (OR), Sensorimotor deficit (PPI)	-

**Table 2:** Effect of GLO1 inhibitors on mouse models for psychiatric disorders.

Authors	Animal model	GLO1 inhibitor	GLO1 activity	MG level		**AGEs**	Effect of GLO1 inhibitor
Distler, et al.[36]	C57BL/6J mouse	↓	↑		-	-	Anxiety↓ (OF)
Distler, et al.[81]	Pilocarpine-treated C57BL/6J mouse	-	-		-	-	Seizure↓
Wang, et al.[38]	Prenatal VPA-treated B6 mouse	↓	↑		-	-	Anxiety↓ (OF, SD, MB), Sociability↑, Nociceptive threshold↓ (TF)
McMurray, et al.[82]	C57BL/6J mouse	-	-		-	-	Alcohol comsumption↓
de Guglielmo, et al.[83]	Wister rat	-	-		-	-	Alcohol self-administration↓
Barkley-Levenson, et al.[84]	FVB/NJ, C57BL/6J mouse	-	-		-	-	HIC after alcohol injection↓
McMurray, et al.[39]	C57BL/6J, BALB/cJ, FVB/NJ mouse	-	-		-	-	Depression↓(TS,FS), Anxiety↓(OF)
	CMS mouse (BALB/cJ)	-	-		-	-	Depression↓(FS)
	OBX mouse (C57BL/6J, BALB/cJ)	-	-		-	-	Hypeactivity↓
Yoshizawa, et al.[40]	C57BL/6N mouse	-	-		-	-	Anxiety↓ (EPM)

Since GLO1 degrades MG, a decrease in GLO1 expression or activity would result in an increase in MG levels in the brain. Consistent with the findings that a decrease in GLO1 expression is associated with a decrease in anxiety, several studies have reported that anxiety is reduced in MG-treated mice [36,42]. The behavioral changes in MG-treated animals are summarized in Table 3. Since the intracerebroventricular administration of MG into the brain also reduces anxiety [43,44], the effect of this MG administration is thought to have a direct effect on the central nervous system. Furthermore, McMurray et al. reported reduced anxiety with the administration of microinjection of MG into the basolateral amygdala, a well-known region of brain responsible for anxiety [37]. Additionally, contrary to the findings so far, several reports have shown that *Glo1* expression is high in mice with low anxiety [[44], [45], [46], [47]]. However, even in these mice, MG levels and MG-derived AGEs were found to be high in the brain [44], indicating that there is a consensus on the relationship between increased MG and reduced anxiety. Regarding the reason why the low-anxiety mice showed high MG levels instead of elevated *Glo1* expression, the authors presumed that the low-anxiety animals were primarily selected for high concentrations of MG and that selection of mice with elevated expression of *Glo1* as well, which is protective against AGE formation, was expected [44]. Thus, the finding is also consistent with those of other studies in that the increase in MG decreases anxiety, although it may contradict them in terms of the amount of *Glo1* expression. This is consistent with our hypothesis that the decrease in GLO1 activity and subsequent increase in MG levels, rather than SNVs and expression within the *GLO1* gene, should be the focus.

**Table 3:** Effect of MG treatment on rodent behavior.

Authors	Rodent model	Treatment	MG treatment	Neurotransmitter	AGE	Behavior	Rescue
Distler, et al.[36]	C57BL/6J mouse	Acute treatment	50, 100, or 300 mg/kg, i.p.	-	-	Anxiety↓ (OF), Locomotor↓, Ataxia↑, Hypothermia↑	-
Distler, et al.[81]	C57BL/6J mouse	Acute treatment	50 or 200 mg/kg, i.p.	-	-	Picrotoxin-induced seizure↓, Pirocarpine-induced seizure↓	-
Szczepanik, et al.[42]	Swiss mouse	Acute treatment	10, 20, or 25 mg/kg, i.p.	-	-	Anxiety↓ (MB, OF), Depression↑ (FS), Long-term spatial memory↓ (OL)	-
		Chronic treatment	10, 20, 25, or 50 mg/kg, i.p. for 5-12 days	DA↓ (PFC), NA n.s. (PFC, HIP), 5-HT n.s. (PFC. HIP)	-	Depression↑ (FS), Long-term memory↓ (OL, OR), Working memory↓ (YM), Long- & short-term aversive memory↓ (IA)	-
de Almeida, et al.[54]	Swiss, C57BL/6, BALB/C mouse	Chronic treatment	25 or 50 mg/kg, i.p. for 7 days	DA↓ (PFC), 5-HT↓ (PFC), DA n.s. (STR), 5-HT n.s. (STR, HIP), NA n.s. (PFC, STR, HIP)	-	Depression↑ (FS), Short-term memory↓ (YM), Long-term spatial memory↓ (OL)	Improved by bupropion
Hansen, et al.[43], Lissner, et al.[89]	Wister rat	ICV administration	3μmol/μl x 1day, 1μmol/μl x 3days, or 0.5μmol/μl x 6days, 5μL, i.c.v.	Glu reuptake↓ (HIP)	CML↑ (HIP)	Anxiety↓ (OF, EPM), Locomotor↓, Long- & short-term memory↓ (OR), Spatial memory↓ (YM)	-
Jakubcakova, et al.[90]	CD1 mouse	ICV administration	0.7μmol, i.c.v.	-	-	Number of NREMS & REMS episode↑, Duration of NREMS & REMS episode↓	-
Hambsch, et al.[44]	CD1 mouse	ICV administration	0.7μmol, i.c.v. for 6 days	-	-	Anxiety↓ (EPM)	-
McMurray, et al.[37]	C57BL/6 mouse	Microinjection into BLA	12μM or 24μM x 0.5uL	-	-	Anxiety↓ (EPM)	-
Yang, et al.[91]	CD1 mouse	Prenatal exposure	0.5mg/kg , i.p. twice daily into pregnant dams (G12/G13 to delivery)	-	-	Social recognition↓, Anxiety↑ (MB)	-

However, how does MG suppress anxiety-like behavior? A recent study provides a possible answer to this question: MG is a partial agonist to the GABA_A_ receptor [36]. It has been electrophysiologically confirmed that MG directly activates the GABA_A_ receptor, leading to the suppression of anxiety-like behavior in mice. These findings suggest that MG can act as an anxiolytic through GABA_A_ receptor activation, similar to benzodiazepine. Thus, it is suggested that high *GLO1* expression can reduce MG concentration in the brain, thereby decreasing the activity of the GABA_A_ receptor, resulting in enhanced anxiety-like behavior.

Contrary to these findings, STZ-treated rats [48,49] and Akita mice [50], which are models of type I diabetes, showed high anxiety with decreased GLO1 expression and accumulation of MG-derived proteins in the brain. In addition, rats that were fed a high-salt diet showed decreased GLO1 expression in the amygdala and hippocampus and enhanced anxiety-like behavior [51]. Moreover, perinatal administration of methylmercury reduces hippocampal expression of GLO1 and enhances anxiety [52]. Various rodent disease models with altered gene expression of *Glo1* are summarized in Table 4. However, there are no data to show that the decrease in *Glo1* expression and subsequent MG accumulation directly affect the enhanced anxiety exhibited in these animal models. We cannot rule out the possibility that anxiety in these animal models was triggered by other factors that had a greater impact than decreased *Glo1* expression. Therefore, to clarify whether *Glo1* expression is involved in anxiety in other disease models, rescue experiments by direct manipulation of *Glo1* gene expression and MG administration would be necessary. Furthermore, measurement of GLO1 activity and quantification of MG levels in the brain are important to clarify the role of GLO1 in these pathological models.

**Table 4:** Rodent models with altered *Glo1* gene expression.

Authors	Animal model		Glo1 expression in brain	GLO1 activity	AGEs	Behavior	Rescue
Karpova, et al.[52]	C57BL/6J mouse	Perinatal methylmercury treatment (G7 to P7)	↓ (HIP), n.s. (PFC)	-	-	Anxiety↑ (EPM, OF), Reversal spatial learning↓ (MWM), Depression↑ (FS)	Improved by TrkB OE
Yang, et al.[55]	SD rat	CUMS for 28 days	↓ (PFC)	-	-	Locomotor↓, Depression↑ (SP)	-
Patki, et al.[56], Solanki, et al.[57]	SD rat	Social defeat stress	↓ (AMY, HIP), n.s. (PFC)	-	-	Anxiety↑ (EPM, LDB, OF, MB), Locomotor↓, Depression ↑ (SP, FS), Long- & short-term memory↓ (RAWM)	Improved by grape powder treatment
Patki, et al.[92]	Wister rat	OVX	↓ (HIP), n.s. (AMY, COR)	-	-	Anxiety↑ (LDB, OF), Short-term memory↓ (RAWM)	Improved by grape powder treatment
Wong, et al.[93]	129S6/SvEvTac mouse	COX2-deficient KI	↓	-	-	Locomotor↑, Anxiety↑ (OF, MB), Motor ability↓ (IST), Sociability↓	-
Zhu, et al.[[48], [49]]	SD rat	Model for type I diabetes by STZ	↓ (COR, HIP, AMY)	-	MG-dependent protein↑ (AMY, HIP)	Depression↑ (FST), Anxiety↑ (OF, EPM)	Improved by treatment of hesperatin or tertbutylhydroquinone
Chugh, et al.[51]	FBN rat	Feeding with high-salt diet	↓ (AMY, HIP), n.s. (COR)	-	-	Anxiety↑ (LDB, OF), Locomotor↓, Short-term memory↓ (RAWM)	-
Maher, et al.[50]	C57BL/6 mouse	Akita mouse model for type I diabetes	↓	↓	MG-dependent protein↑ (COR)	Locomotor↓	Improved by fisetin treatment

↑: Increase, ↓: Decrease, -: Not checked, n.s.: No significant, SD: Sprague-Dawley, FBN: Fischer brown Norway, OVX: ovariectomized, KI: Knock-in, CUMS: chronic unpredictable mild stress, OE: Overexpression, AMY: Amygdala, HIP: Hippocampus, PFC: Prefrontal cortex, COR: Cortex, OF: Open field test, TS: Tail suspension test, FS: Forced-swimming test, MWM: Morris water maze test, SP: Sucrose preference test, MB: Marble burying test, EPM: Elevated plus-maze test, LDB: Light and dark test, RAWM: Radial-arm water maze test, IST: Inverted screen test

### 3.2 Depression

GLO1 inhibitors have been reported to be useful as novel antidepressants [39]. Two structurally distinct GLO1 inhibitors (*S*-bromobenzylglutathione cyclopentyl diester or methyl-gerfelin) ameliorated depression-like behavior in a rodent model after 5 days of treatment, whereas an existing antidepressant, fluoxetine, takes 14 days of treatment to ameliorate depression-like behavior. Furthermore, the 5-day treatment with GLO1 inhibitors induced molecular markers of the antidepressant response, including brain-derived neurotrophic factor and cyclic-AMP response-binding protein phosphorylation in the hippocampus and medial prefrontal cortex. These findings are also supported by a report showing that *Glo1* is one of the genes contributing most to the antidepressant response [53]. Thus, GLO1 inhibitors may be effective as novel and fast-acting drugs for treating depression.

In contrast, several reports have shown that MG-treated mice exhibit depression-like behavior [42,54]. In addition, rodent models of depression induced by chronic mild stress [55] and social defeat stress [56,57] showed depression-like behaviors with decreased GLO1 expression in the brain. Furthermore, we have shown that *Glo1* KO mice showed no accumulation of MG in the brain, although *Glo1* KO mice exhibit antidepressant-like behavior [58]. This result is supported by *in vitro* and *in vivo* studies showing that in the absence of GLO1, aldo-keto reductase may compensate for MG detoxification, resulting in no MG accumulation [9,10]. Another group also reported that *Glo1* KO induced no accumulation of MG-H1, an MG-derived AGE, in the brain [41].

Thus, further studies are required to evaluate the pharmacological mechanisms underlying the antidepressant effects of GLO1 inhibitors. For example, the molecular mechanism underlying the anxiolytic and antidepressant-like effect of GLO1 inhibitors is thought to be due to GABA_A_ receptor stimulation by accumulated MG [39], but there is still no direct evidence that decreased GLO1 activity actually stimulates the GABAergic system in the brain, including the hippocampus and prefrontal cortex, through MG accumulation, leading to low levels of anxiety. In addition, since a previous study reported that *Glo1* KO mice showed antidepressant-like behavior in the forced swimming test even though there was no MG accumulation [58], it is necessary to examine how GLO inhibitors could reduce MG levels in the brain. We believe that it is also necessary to consider the possibility that the administration of GLO1 inhibitors improved depression-like behavior without the pharmacological effects of MG.

### 3.3 Schizophrenia

As mentioned above, we have reported that several patients with schizophrenia have a novel frameshift mutation and SNP in *GLO1* that results in reductions in enzymatic activity [22]. To elucidate the impact of loss of function of GLO1 on the pathogenesis of schizophrenia, we generated *Glo1* KO mice and evaluated their behavioral phenotypes [58]. However, the *Glo1* KO mice did not show schizophrenia-like behavior, and the MG levels in the brain remained unchanged.

Next, we sought to develop a novel model for schizophrenia with an add-on treatment of an environmental factor, vitamin B6 (VB6) deficiency, observed in patients with schizophrenia [22,59,60], in *Glo1* KO mice. VB6 is known as a carbonyl scavenger that detoxifies MG [61,62]. We previously found that VB6 levels in the peripheral blood of schizophrenia patients with *GLO1* dysfunction were significantly lower than those in healthy controls [22]. More than 35% of patients with schizophrenia have low levels of VB6 (clinically defined as male, < 6 ng/ml; female, < 4 ng/ml). Various other studies have reported similar results [63,64]. VB6 levels are inversely proportional to the severity score on the Positive and Negative Syndrome Scale [60]. Moreover, a recent review has shown that decreased VB6 in patients with schizophrenia is the most convincing evidence of peripheral biomarkers for major mental disorders [65]. Moreover, we recently reported that VB6-deficient mice showed an enhanced noradrenergic system in the brain, leading to social deficits and cognitive impairment comparable to the negative symptoms and cognitive impairment seen in patients with schizophrenia [66].

Based on these findings, we depleted VB6 in *Glo1* KO mice by feeding them VB6-lacking diets to develop a mouse model that further recapitulates the pathology of schizophrenia. We demonstrated that MG accumulated in the brain of *Glo1* KO mice with VB6 deficiency (KO/VB6(−)), and that these mice exhibited schizophrenia-like behaviors, such as a sensorimotor deficit in the prepulse inhibition test [58]. These findings suggest that *Glo1* deletion alone is insufficient to increase MG levels in the brain, and MG accumulation in the brain occurs only when another MG detoxification is deficit, such as VB6 deficiency. In fact, we could also confirm the accumulation of MG-H1, an MG-derived AGE, in the brains of KO/VB6(−) mice [67].

Furthermore, we found aberrant gene expression related to mitochondrial function and respiratory deficits in the mitochondria of KO/VB6(−) mice. Moreover, we found higher expression of oxidative stress markers as a result of mitochondrial dysfunction. MG can also disrupt mitochondrial respiration; incubation of isolated mitochondria with MG produced a concentration-dependent decrease in state III, as well as an increase and then a decrease in state IV respiration [68]. These findings suggest that mitochondrial dysfunction can be caused by aberrant gene expression related to mitochondria and MG accumulation. Our findings are consistent with the result that *GLO1* KO-hiPSC-derived neurons show mitochondrial dysfunction and MG-H1 accumulation after MG treatment [69].

Mitochondrial dysfunction has frequently been reported in patients with schizophrenia [70]. Several studies using postmortem brains demonstrated a decrease in the activity of complex IV in the PFC of patients with schizophrenia [71], and a global downregulation of mitochondria-related genes by microarray analysis [72]. Mitochondrial dysfunction has also been observed in patient-derived iPS cells [73,74]. These findings suggest that the combination of GLO1 dysfunction and VB6 deficiency may affect mitochondrial function in KO/VB6(−) mice, causing schizophrenia-like sensorimotor deficits. Mitochondrial dysfunction has been observed in many psychiatric disorders, including depression [75], autism [76], anxiety disorders [77], and epilepsy [78]. Thus, this may be a common molecular basis for psychiatric disorders.

In a mouse model of schizophrenia, as well as other physical disorders, MG accumulation was found to have adverse effects on brain function. Considering the molecular mechanism revealed in this study, GLO1 inducers, such as *trans*-resveratrol and hesperatin [79], rather than inhibitors and VB6 supplementation [80] may be effective as a new therapeutic strategy for schizophrenia patients with GLO1 dysfunction and VB6 deficiency.

### 3.4 Other psychiatric and neurological traits

A recent study on prenatal treatment with valproic acid (VPA) in a mouse model of autism showed enhanced GLO1 expression and reduced MG levels in the brain [38]. Furthermore, GLO1 inhibitors ameliorated the autism-like phenotype in VPA-treated mice through GABA_A_ stimulation by MG. These findings are not consistent with the clinical findings that GLO1 enzymatic activity was decreased, and AGE accumulation was found in the autopsied brains of patients with autism [14]. The reason for this disagreement may be secondary effects unrelated to the autistic pathology caused by prenatal VPA administration. In addition, the possibility that the decreased GLO1 activity observed in patients with autism reflects secondary effects of the onset of autism may need to be considered.

The GABA_A_ agonistic activity of MG has also been reported to have antiepileptic and alcohol detoxification effects, such as benzodiazepine. Pretreatment with MG attenuated picrotoxin- and pilocarpine-induced seizures at both behavioral and electroencephalogram levels [81]. Pretreatment with a GLO1 inhibitor also alleviated pilocarpine-induced seizures, whereas *Glo1* overexpression exacerbated seizures and decreased the MG concentration in the brain. Likewise, GLO1 inhibitors reduced alcohol consumption [82,83] and handling-induced convulsion after alcohol injection [84], whereas *Glo1* overexpression exacerbated these effects. In addition, alcohol consumption was reduced in *Glo1* hemizygous-knockdown mice [82]. Although there are no clinical reports showing an association between alcoholism/sleep disorder and *GLO1*, a clinical study reported that *rs1049346* T>C SNP in 5′-UTR of *GLO1* was associated with late-onset epilepsy and drug-resistant epilepsy [85]. The C allele increases GLO1 enzymatic activity in whole blood cells [86]. These findings are consistent with the results of rodent studies.

## 4 Summary

According to the present review, the expression and activity of GLO1 in the brain may be involved in the pathogenesis of various psychiatric disorders. In contrast to the effects on physical diseases, the various GABA_A_ receptor-mediated effects of MG, such as anxiolytic and antidepressant effects, associated with suppression of GLO1 function, are brain-specific effects. To clarify the effects of GLO1 on brain function and the development of psychiatric disorders, it is necessary to clarify the relationship between GLO1 activity and psychiatric disorders in clinical studies with large sample sizes and to examine in detail the molecular mechanisms by which increased GLO1 activity leads to anxiety and depression in studies involving mouse models.

## Funding and disclosure

This work was supported by 10.13039/501100001691JSPS KAKENHI Grant Numbers 16H05380, 17H05930, 18K06977, 19H04887, 20H03608; 10.13039/100009619AMED under Grant Number JP20dm0107088, the 10.13039/501100008880Kanae Foundation for the Promotion of Medical Science, the 10.13039/100008732Uehara Memorial Foundation, 10.13039/100008608The Sumitomo Foundation, and the 10.13039/501100008667SENSHIN Medical Research Foundation, 10.13039/100007449Takeda Science Foundation.

## Declaration of competing interest

The authors have declared that no conflict of interest exists.

## References

[1] Rabbani N., Thornalley P.J. (2015). Dicarbonyl stress in cell and tissue dysfunction contributing to ageing and disease. Biochem. Biophys. Res. Commun..

[2] Hipkiss A.R. (2017). On the relationship between energy metabolism, proteostasis, aging and Parkinson's disease: possible causative role of methylglyoxal and alleviative potential of carnosine. Aging Dis..

[3] Rabbani N., Xue M., Thornalley P.J. (2016). Methylglyoxal-induced dicarbonyl stress in aging and disease: first steps towards glyoxalase 1-based treatments. Clin. Sci. (Lond.).

[4] Thornalley P.J. (2007). Endogenous alpha-oxoaldehydes and formation of protein and nucleotide advanced glycation endproducts in tissue damage. Novartis Found. Symp..

[5] Valente T., Gella A., Fernandez-Busquets X., Unzeta M., Durany N. (2010). Immunohistochemical analysis of human brain suggests pathological synergism of Alzheimer's disease and diabetes mellitus. Neurobiol. Dis..

[6] Ramasamy R., Yan S.F., Schmidt A.M. (2012). Advanced glycation endproducts: from precursors to RAGE: round and round we go. Amino Acids.

[7] Mannervik B. (2008). Molecular enzymology of the glyoxalase system. Drug Metabol. Drug Interact..

[8] Lee J.Y., Song J., Kwon K., Jang S., Kim C., Baek K., Kim J., Park C. (2012). Human DJ-1 and its homologs are novel glyoxalases. Hum. Mol. Genet..

[9] Morgenstern J., Fleming T., Schumacher D., Eckstein V., Freichel M., Herzig S., Nawroth P. (2017). Loss of glyoxalase 1 induces compensatory mechanism to achieve dicarbonyl detoxification in mammalian schwann cells. J. Biol. Chem..

[10] Schumacher D., Morgenstern J., Oguchi Y., Volk N., Kopf S., Groener J.B., Nawroth P.P., Fleming T., Freichel M. (2018). Compensatory mechanisms for methylglyoxal detoxification in experimental & clinical diabetes. Mol. Metabol..

[11] Vander Jagt D.L., Hunsaker L.A. (2003). Methylglyoxal metabolism and diabetic complications: roles of aldose reductase, glyoxalase-I, betaine aldehyde dehydrogenase and 2-oxoaldehyde dehydrogenase. Chem. Biol. Interact..

[12] Vander Jagt D.L., Robinson B., Taylor K.K., Hunsaker L.A. (1992). Reduction of trioses by NADPH-dependent aldo-keto reductases. Aldose reductase, methylglyoxal, and diabetic complications. J. Biol. Chem..

[13] Politi P., Minoretti P., Falcone C., Martinelli V., Emanuele E. (2006). Association analysis of the functional Ala111Glu polymorphism of the glyoxalase I gene in panic disorder. Neurosci. Lett..

[14] Junaid M.A., Kowal D., Barua M., Pullarkat P.S., Sklower Brooks S., Pullarkat R.K. (2004). Proteomic studies identified a single nucleotide polymorphism in glyoxalase I as autism susceptibility factor. Am. J. Med. Genet..

[15] Kovac J., Podkrajsek K.T., Luksic M.M., Battelino T. (2015). Weak association of glyoxalase 1 (GLO1) variants with autism spectrum disorder. Eur. Child Adolesc. Psychiatr..

[16] Barua M., Jenkins E.C., Chen W., Kuizon S., Pullarkat R.K., Junaid M.A. (2011). Glyoxalase I polymorphism rs2736654 causing the Ala111Glu substitution modulates enzyme activity-implications for autism. Autism Res..

[17] Nembaware V., Lupindo B., Schouest K., Spillane C., Scheffler K., Seoighe C. (2008). Genome-wide survey of allele-specific splicing in humans. BMC Genom..

[18] Wu Y.Y., Chien W.H., Huang Y.S., Gau S.S., Chen C.H. (2008). Lack of evidence to support the glyoxalase 1 gene (GLO1) as a risk gene of autism in Han Chinese patients from Taiwan. Prog. Neuro-Psychopharmacol. Biol. Psychiatry.

[19] Sacco R., Papaleo V., Hager J., Rousseau F., Moessner R., Militerni R., Bravaccio C., Trillo S., Schneider C., Melmed R., Elia M., Curatolo P., Manzi B., Pascucci T., Puglisi-Allegra S., Reichelt K.L., Persico A.M. (2007). Case-control and family-based association studies of candidate genes in autistic disorder and its endophenotypes: TPH2 and GLO1. BMC Med. Genet..

[20] Rehnstrom K., Ylisaukko-Oja T., Vanhala R., von Wendt L., Peltonen L., Hovatta I. (2008). No association between common variants in glyoxalase 1 and autism spectrum disorders. Am. J. Med. Genet. B Neuropsychiatr. Genet..

[21] Gabriele S., Lombardi F., Sacco R., Napolioni V., Altieri L., Tirindelli M.C., Gregorj C., Bravaccio C., Rousseau F., Persico A.M. (2014). The GLO1 C332 (Ala111) allele confers autism vulnerability: family-based genetic association and functional correlates. J. Psychiatr. Res..

[22] Arai M., Yuzawa H., Nohara I., Ohnishi T., Obata N., Iwayama Y., Haga S., Toyota T., Ujike H., Arai M., Ichikawa T., Nishida A., Tanaka Y., Furukawa A., Aikawa Y., Kuroda O., Niizato K., Izawa R., Nakamura K., Mori N., Matsuzawa D., Hashimoto K., Iyo M., Sora I., Matsushita M., Okazaki Y., Yoshikawa T., Miyata T., Itokawa M. (2010). Enhanced carbonyl stress in a subpopulation of schizophrenia. Arch. Gen. Psychiatr..

[23] Ishizuka K., Kimura H., Kushima I., Inada T., Okahisa Y., Ikeda M., Iwata N., Mori D., Aleksic B., Ozaki N. (2018). Assessment of a glyoxalase I frameshift variant, p.P122fs, in Japanese patients with schizophrenia. Psychiatr. Genet..

[24] Mizutani R., Saiga R., Takeuchi A., Uesugi K., Terada Y., Suzuki Y., De Andrade V., De Carlo F., Takekoshi S., Inomoto C., Nakamura N., Kushima I., Iritani S., Ozaki N., Ide S., Ikeda K., Oshima K., Itokawa M., Arai M. (2019). Three-dimensional alteration of neurites in schizophrenia. Transl. Psychiatry.

[25] Yin J., Ma G., Luo S., Luo X., He B., Liang C., Zuo X., Xu X., Chen Q., Xiong S., Tan Z., Fu J., Lv D., Dai Z., Wen X., Zhu D., Ye X., Lin Z., Lin J., Li Y., Chen W., Luo Z., Li K., Wang Y. (2021). Glyoxalase 1 confers susceptibility to schizophrenia: from genetic variants to phenotypes of neural function. Front. Mol. Neurosci..

[26] Yoshioka S., Odani H., Furuhashi T., Tanaka T., Ogawa T. (2021). Increase in the peripheral blood methylglyoxal levels in 10% of hospitalized chronic schizophrenia patients. Nagoya J. Med. Sci..

[27] Fujimoto M., Uchida S., Watanuki T., Wakabayashi Y., Otsuki K., Matsubara T., Suetsugi M., Funato H., Watanabe Y. (2008). Reduced expression of glyoxalase-1 mRNA in mood disorder patients. Neurosci. Lett..

[28] Hall L.S., Adams M.J., Arnau-Soler A., Clarke T.K., Howard D.M., Zeng Y., Davies G., Hagenaars S.P., Maria Fernandez-Pujals A., Gibson J., Wigmore E.M., Boutin T.S., Hayward C., Scotland G., Major Depressive Disorder Working Group of the Psychiatric Genomics C., Porteous D.J., Deary I.J., Thomson P.A., Haley C.S., McIntosh A.M. (2018). Genome-wide meta-analyses of stratified depression in generation scotland and UK biobank. Transl. Psychiatry.

[29] Warrier V., Chee V., Smith P., Chakrabarti B., Baron-Cohen S. (2015). A comprehensive meta-analysis of common genetic variants in autism spectrum conditions. Mol. Autism..

[30] Cameron A.D., Olin B., Ridderstrom M., Mannervik B., Jones T.A. (1997). Crystal structure of human glyoxalase I--evidence for gene duplication and 3D domain swapping. EMBO J..

[31] Morgenstern J., Katz S., Krebs-Haupenthal J., Chen J., Saadatmand A., Cortizo F.G., Moraru A., Zemva J., Campos M.C., Teleman A., Backs J., Nawroth P., Fleming T. (2020). Phosphorylation of T107 by CamKIIdelta regulates the detoxification efficiency and proteomic integrity of glyoxalase 1. Cell Rep..

[32] Hovatta I., Tennant R.S., Helton R., Marr R.A., Singer O., Redwine J.M., Ellison J.A., Schadt E.E., Verma I.M., Lockhart D.J., Barlow C. (2005). Glyoxalase 1 and glutathione reductase 1 regulate anxiety in mice. Nature.

[33] Szego E.M., Janaky T., Szabo Z., Csorba A., Kompagne H., Muller G., Levay G., Simor A., Juhasz G., Kekesi K.A. (2010). A mouse model of anxiety molecularly characterized by altered protein networks in the brain proteome. Eur. Neuropsychopharmacol.

[34] Matsuo K., Watanabe T., Takenaka A. (2019). Effect of dietary vitamin E on oxidative stress-related gene-mediated differences in anxiety-like behavior in inbred strains of mice. Physiol. Behav..

[35] Williams R.t., Lim J.E., Harr B., Wing C., Walters R., Distler M.G., Teschke M., Wu C., Wiltshire T., Su A.I., Sokoloff G., Tarantino L.M., Borevitz J.O., Palmer A.A. (2009). A common and unstable copy number variant is associated with differences in Glo1 expression and anxiety-like behavior. PLoS One.

[36] Distler M.G., Plant L.D., Sokoloff G., Hawk A.J., Aneas I., Wuenschell G.E., Termini J., Meredith S.C., Nobrega M.A., Palmer A.A. (2012). Glyoxalase 1 increases anxiety by reducing GABAA receptor agonist methylglyoxal. J. Clin. Invest..

[37] McMurray K.M., Du X., Brownlee M., Palmer A.A. (2016). Neuronal overexpression of Glo1 or amygdalar microinjection of methylglyoxal is sufficient to regulate anxiety-like behavior in mice. Behav. Brain Res..

[38] Wang K., Li N., Xu M., Huang M., Huang F. (2020). Glyoxalase 1 inhibitor alleviates autism-like phenotype in a prenatal valproic acid-induced mouse model. ACS Chem. Neurosci..

[39] McMurray K.M.J., Ramaker M.J., Barkley-Levenson A.M., Sidhu P.S., Elkin P.K., Reddy M.K., Guthrie M.L., Cook J.M., Rawal V.H., Arnold L.A., Dulawa S.C., Palmer A.A. (2018). Identification of a novel, fast-acting GABAergic antidepressant. Mol. Psychiatr..

[40] Yoshizawa K., Tabuchi M., Ukai S., Suzuki H., Kawano Y., Takasawa R. (2020). The putative glyoxalase 1 inhibitor piceatannol exhibits both anxiolytic-like and antitumor effects in mice. Anticancer Res..

[41] Jang S., Kwon D.M., Kwon K., Park C. (2017). Generation and characterization of mouse knockout for glyoxalase 1. Biochem. Biophys. Res. Commun..

[42] Szczepanik J.C., de Almeida G.R.L., Cunha M.P., Dafre A.L. (2020). Repeated methylglyoxal treatment depletes dopamine in the prefrontal cortex, and causes memory impairment and depressive-like behavior in mice. Neurochem. Res..

[43] Hansen F., Pandolfo P., Galland F., Torres F.V., Dutra M.F., Batassini C., Guerra M.C., Leite M.C., Goncalves C.A. (2016). Methylglyoxal can mediate behavioral and neurochemical alterations in rat brain. Physiol. Behav..

[44] Hambsch B., Chen B.G., Brenndorfer J., Meyer M., Avrabos C., Maccarrone G., Liu R.H., Eder M., Turck C.W., Landgraf R. (2010). Methylglyoxal-mediated anxiolysis involves increased protein modification and elevated expression of glyoxalase 1 in the brain. J. Neurochem..

[45] Kromer S.A., Kessler M.S., Milfay D., Birg I.N., Bunck M., Czibere L., Panhuysen M., Putz B., Deussing J.M., Holsboer F., Landgraf R., Turck C.W. (2005). Identification of glyoxalase-I as a protein marker in a mouse model of extremes in trait anxiety. J. Neurosci..

[46] Ditzen C., Jastorff A.M., Kessler M.S., Bunck M., Teplytska L., Erhardt A., Kromer S.A., Varadarajulu J., Targosz B.S., Sayan-Ayata E.F., Holsboer F., Landgraf R., Turck C.W. (2006). Protein biomarkers in a mouse model of extremes in trait anxiety. Mol. Cell. Proteomics.

[47] Zhang Y., Filiou M.D., Reckow S., Gormanns P., Maccarrone G., Kessler M.S., Frank E., Hambsch B., Holsboer F., Landgraf R., Turck C.W. (2011). Proteomic and metabolomic profiling of a trait anxiety mouse model implicate affected pathways. Mol. Cell. Proteomics.

[48] Zhu X., Zhang Y.M., Zhang M.Y., Chen Y.J., Liu Y.W. (2021). Hesperetin ameliorates diabetes-associated anxiety and depression-like behaviors in rats via activating Nrf2/ARE pathway. Metab. Brain Dis..

[49] Zhu X., Liu H., Liu Y., Chen Y., Liu Y., Yin X. (2020). The antidepressant-like effects of hesperidin in streptozotocin-induced diabetic rats by activating Nrf2/ARE/glyoxalase 1 pathway. Front. Pharmacol..

[50] Maher P., Dargusch R., Ehren J.L., Okada S., Sharma K., Schubert D. (2011). Fisetin lowers methylglyoxal dependent protein glycation and limits the complications of diabetes. PLoS One.

[51] Chugh G., Asghar M., Patki G., Bohat R., Jafri F., Allam F., Dao A.T., Mowrey C., Alkadhi K., Salim S. (2013). A high-salt diet further impairs age-associated declines in cognitive, behavioral, and cardiovascular functions in male Fischer brown Norway rats. J. Nutr..

[52] Karpova N.N., Lindholm J.S., Kulesskaya N., Onishchenko N., Vahter M., Popova D., Ceccatelli S., Castren E. (2014). TrkB overexpression in mice buffers against memory deficits and depression-like behavior but not all anxiety- and stress-related symptoms induced by developmental exposure to methylmercury. Front. Behav. Neurosci..

[53] Benton C.S., Miller B.H., Skwerer S., Suzuki O., Schultz L.E., Cameron M.D., Marron J.S., Pletcher M.T., Wiltshire T. (2012). Evaluating genetic markers and neurobiochemical analytes for fluoxetine response using a panel of mouse inbred strains. Psychopharmacology (Berl).

[54] de Almeida G.R.L., Szczepanik J.C., Selhorst I., Schmitz A.E., Dos Santos B., Cunha M.P., Heinrich I.A., de Paula G.C., De Bem A.F., Leal R.B., Dafre A.L. (2021). Methylglyoxal-mediated dopamine depletion, working memory deficit, and depression-like behavior are prevented by a dopamine/noradrenaline reuptake inhibitor. Mol. Neurobiol..

[55] Yang Y., Yang D., Tang G., Zhou C., Cheng K., Zhou J., Wu B., Peng Y., Liu C., Zhan Y., Chen J., Chen G., Xie P. (2013). Proteomics reveals energy and glutathione metabolic dysregulation in the prefrontal cortex of a rat model of depression. Neuroscience.

[56] Patki G., Solanki N., Atrooz F., Allam F., Salim S. (2013). Depression, anxiety-like behavior and memory impairment are associated with increased oxidative stress and inflammation in a rat model of social stress. Brain Res..

[57] Solanki N., Salvi A., Patki G., Salim S. (2017). Modulating oxidative stress relieves stress-induced behavioral and cognitive impairments in rats. Int. J. Neuropsychopharmacol..

[58] Toriumi K., Berto S., Koike S., Usui N., Dan T., Suzuki K., Miyashita M., Horiuchi Y., Yoshikawa A., Asakura M., Nagahama K., Lin H.C., Sugaya Y., Watanabe T., Kano M., Ogasawara Y., Miyata T., Itokawa M., Konopka G., Arai M. (2021). Combined glyoxalase 1 dysfunction and vitamin B6 deficiency in a schizophrenia model system causes mitochondrial dysfunction in the prefrontal cortex. Redox Biol..

[59] Arai M., Miyashita M., Kobori A., Toriumi K., Horiuchi Y., Itokawa M. (2014). Carbonyl stress and schizophrenia. Psychiatr. Clin. Neurosci..

[60] Miyashita M., Arai M., Kobori A., Ichikawa T., Toriumi K., Niizato K., Oshima K., Okazaki Y., Yoshikawa T., Amano N., Miyata T., Itokawa M. (2014). Clinical features of schizophrenia with enhanced carbonyl stress. Schizophr. Bull..

[61] Onorato J.M., Jenkins A.J., Thorpe S.R., Baynes J.W. (2000). Pyridoxamine, an inhibitor of advanced glycation reactions, also inhibits advanced lipoxidation reactions. Mechanism of action of pyridoxamine. J. Biol. Chem..

[62] Amarnath V., Amarnath K., Amarnath K., Davies S., Roberts L.J. (2004). 2nd, Pyridoxamine: an extremely potent scavenger of 1,4-dicarbonyls. Chem. Res. Toxicol..

[63] Katsuta N., Ohnuma T., Maeshima H., Takebayashi Y., Higa M., Takeda M., Nakamura T., Nishimon S., Sannohe T., Hotta Y., Hanzawa R., Higashiyama R., Shibata N., Arai H. (2014). Significance of measurements of peripheral carbonyl stress markers in a cross-sectional and longitudinal study in patients with acute-stage schizophrenia. Schizophr. Bull..

[64] Tomioka Y., Numata S., Kinoshita M., Umehara H., Watanabe S.-y., Nakataki M., Iwayama Y., Toyota T., Ikeda M., Yamamori H., Shimodera S., Tajima A., Hashimoto R., Iwata N., Yoshikawa T., Ohmori T. (2018). Decreased serum pyridoxal levels in schizophrenia: meta-analysis and Mendelian randomization analysis. J. Psychiatry Neurosci..

[65] Carvalho A.F., Solmi M., Sanches M., Machado M.O., Stubbs B., Ajnakina O., Sherman C., Sun Y.R., Liu C.S., Brunoni A.R., Pigato G., Fernandes B.S., Bortolato B., Husain M.I., Dragioti E., Firth J., Cosco T.D., Maes M., Berk M., Lanctot K.L., Vieta E., Pizzagalli D.A., Smith L., Fusar-Poli P., Kurdyak P.A., Fornaro M., Rehm J., Herrmann N. (2020). Evidence-based umbrella review of 162 peripheral biomarkers for major mental disorders. Transl. Psychiatry.

[66] Toriumi K., Miyashita M., Suzuki K., Yamasaki N., Yasumura M., Horiuchi Y., Yoshikawa A., Asakura M., Usui N., Itokawa M., Arai M. (2021). Vitamin B6 deficiency hyperactivates the noradrenergic system, leading to social deficits and cognitive impairment. Transl. Psychiatry.

[67] Koike S., Toriumi K., Kasahara S., Kibune Y., Ishida Y.I., Dan T., Miyata T., Arai M., Ogasawara Y. (2021). Accumulation of carbonyl proteins in the brain of mouse model for methylglyoxal detoxification deficits. Antioxidants.

[68] Rosca M.G., Monnier V.M., Szweda L.I., Weiss M.F. (2002). Alterations in renal mitochondrial respiration in response to the reactive oxoaldehyde methylglyoxal. Am. J. Physiol. Ren. Physiol..

[69] Hara T., Toyoshima M., Hisano Y., Balan S., Iwayama Y., Aono H., Futamura Y., Osada H., Owada Y., Yoshikawa T. (2021). Glyoxalase I disruption and external carbonyl stress impair mitochondrial function in human induced pluripotent stem cells and derived neurons. Transl. Psychiatry.

[70] Ben-Shachar D., Laifenfeld D. (2004). Mitochondria, synaptic plasticity, and schizophrenia. Int. Rev. Neurobiol..

[71] Maurer I., Zierz S., Moller H. (2001). Evidence for a mitochondrial oxidative phosphorylation defect in brains from patients with schizophrenia. Schizophr. Res..

[72] Ni P., Noh H., Park G.H., Shao Z., Guan Y., Park J.M., Yu S., Park J.S., Coyle J.T., Weinberger D.R., Straub R.E., Cohen B.M., McPhie D.L., Yin C., Huang W., Kim H.Y., Chung S. (2020). iPSC-derived homogeneous populations of developing schizophrenia cortical interneurons have compromised mitochondrial function. Mol. Psychiatr..

[73] Iwamoto K., Bundo M., Kato T. (2005). Altered expression of mitochondria-related genes in postmortem brains of patients with bipolar disorder or schizophrenia, as revealed by large-scale DNA microarray analysis. Hum. Mol. Genet..

[74] Robicsek O., Karry R., Petit I., Salman-Kesner N., Muller F.J., Klein E., Aberdam D., Ben-Shachar D. (2013). Abnormal neuronal differentiation and mitochondrial dysfunction in hair follicle-derived induced pluripotent stem cells of schizophrenia patients. Mol. Psychiatr..

[75] Karabatsiakis A., Schonfeldt-Lecuona C. (2020). Depression, mitochondrial bioenergetics, and electroconvulsive therapy: a new approach towards personalized medicine in psychiatric treatment - a short review and current perspective. Transl. Psychiatry.

[76] Rossignol D.A., Frye R.E. (2012). Mitochondrial dysfunction in autism spectrum disorders: a systematic review and meta-analysis. Mol. Psychiatr..

[77] Hollis F., van der Kooij M.A., Zanoletti O., Lozano L., Canto C., Sandi C. (2015). Mitochondrial function in the brain links anxiety with social subordination. Proc. Natl. Acad. Sci. U. S. A..

[78] Rahman S. (2012). Mitochondrial disease and epilepsy. Dev. Med. Child Neurol..

[79] Xue M., Weickert M.O., Qureshi S., Kandala N.B., Anwar A., Waldron M., Shafie A., Messenger D., Fowler M., Jenkins G., Rabbani N., Thornalley P.J. (2016). Improved glycemic control and vascular function in overweight and obese subjects by glyoxalase 1 inducer formulation. Diabetes.

[80] Itokawa M., Miyashita M., Arai M., Dan T., Takahashi K., Tokunaga T., Ishimoto K., Toriumi K., Ichikawa T., Horiuchi Y., Kobori A., Usami S., Yoshikawa T., Amano N., Washizuka S., Okazaki Y., Miyata T. (2018). Pyridoxamine: a novel treatment for schizophrenia with enhanced carbonyl stress. Psychiatr. Clin. Neurosci..

[81] Distler M.G., Gorfinkle N., Papale L.A., Wuenschell G.E., Termini J., Escayg A., Winawer M.R., Palmer A.A. (2013). Glyoxalase 1 and its substrate methylglyoxal are novel regulators of seizure susceptibility. Epilepsia.

[82] McMurray K.M., Sidhu P.S., Cook J.M., Arnold L.A., Palmer A.A. (2017). Genetic and pharmacological manipulation of glyoxalase 1 regulates voluntary ethanol consumption in mice. Addiction Biol..

[83] de Guglielmo G., Conlisk D.E., Barkley-Levenson A.M., Palmer A.A., George O. (2018). Inhibition of Glyoxalase 1 reduces alcohol self-administration in dependent and nondependent rats. Pharmacol. Biochem. Behav..

[84] Barkley-Levenson A.M., Lee A., Palmer A.A. (2021). Genetic and pharmacological manipulations of glyoxalase 1 mediate ethanol withdrawal seizure susceptibility in mice. Brain Sci..

[85] Tao H., Si L., Zhou X., Liu Z., Ma Z., Zhou H., Zhong W., Cui L., Zhang S., Li Y., Ma G., Zhao J., Huang W., Yao L., Xu Z., Zhao B., Li K. (2016). Role of glyoxalase I gene polymorphisms in late-onset epilepsy and drug-resistant epilepsy. J. Neurol. Sci..

[86] Peculis R., Konrade I., Skapare E., Fridmanis D., Nikitina-Zake L., Lejnieks A., Pirags V., Dambrova M., Klovins J. (2013). Identification of glyoxalase 1 polymorphisms associated with enzyme activity. Gene.

[87] Hambsch B. (2011). Altered glyoxalase 1 expression in psychiatric disorders: cause or consequence?. Semin. Cell Dev. Biol..

[88] El-Osta A., Brasacchio D., Yao D., Pocai A., Jones P.L., Roeder R.G., Cooper M.E., Brownlee M. (2008). Transient high glucose causes persistent epigenetic changes and altered gene expression during subsequent normoglycemia. J. Exp. Med..

[89] Lissner L.J., Rodrigues L., Wartchow K.M., Borba E., Bobermin L.D., Fontella F.U., Hansen F., Quincozes-Santos A., Souza D.O.G., Goncalves C.A. (2021). Short-term alterations in behavior and astroglial function after intracerebroventricular infusion of methylglyoxal in rats. Neurochem. Res..

[90] Jakubcakova V., Curzi M.L., Flachskamm C., Hambsch B., Landgraf R., Kimura M. (2013). The glycolytic metabolite methylglyoxal induces changes in vigilance by generating low-amplitude non-REM sleep. J. Psychopharmacol..

[91] Yang G., Cancino G.I., Zahr S.K., Guskjolen A., Voronova A., Gallagher D., Frankland P.W., Kaplan D.R., Miller F.D. (2016). A glo1-methylglyoxal pathway that is perturbed in maternal diabetes regulates embryonic and adult neural stem cell pools in murine offspring. Cell Rep..

[92] Patki G., Allam F.H., Atrooz F., Dao A.T., Solanki N., Chugh G., Asghar M., Jafri F., Bohat R., Alkadhi K.A., Salim S. (2013). Grape powder intake prevents ovariectomy-induced anxiety-like behavior, memory impairment and high blood pressure in female Wistar rats. PLoS One.

[93] Wong C.T., Bestard-Lorigados I., Crawford D.A. (2019). Autism-related behaviors in the cyclooxygenase-2-deficient mouse model. Gene Brain Behav..

